# Functional evaluation of cyclosporine metabolism by CYP3A4 variants and potential drug interactions

**DOI:** 10.3389/fphar.2022.1044817

**Published:** 2023-01-06

**Authors:** Qihui Kong, Nanyong Gao, Yahui Wang, Guoxin Hu, Jianchang Qian, Bingbing Chen

**Affiliations:** Institute of Molecular Toxicology and Pharmacology, School of Pharmaceutical Sciences, Wenzhou Medical University, Wenzhou, Zhejiang, China

**Keywords:** CsA, cyclosporine, nimodipine, CYP3A4, polymorphisms, interaction

## Abstract

The aim of this study is to investigate the effects of CYP3A4 genetic polymorphisms on the metabolism of cyclosporine (CsA) *in vitro* and identify drugs that interact with CsA. An enzymatic incubation system was developed to evaluate the kinetic parameters of CYP3A4 on CsA catalysis. A total of 132 drugs were screened to identify potential drug–drug interactions. Sprague–Dawley rats were used to determine the interaction between CsA and nimodipine and nisoldipine. The metabolite AM1 was measured by ultra-performance liquid chromatography–tandem mass spectrometry. The results demonstrate that 16 CYP3A4 variants (CYP3A4.7, 8, 9, 12, 13, 14, 16, 18, 19, 23, 24, 28, 31, 32, 33, and 34) have a lower metabolic capacity for CsA, ranging from 7.19% to 72.10%, than CYP3A4.1. In contrast, the relative clearance rate of CYP3A4.5 is significantly higher than that of CYP3A4.1. Moreover, CYP3A4.20 loses its catalytic ability, and five other variants have no significant difference. A total of 12 drugs, especially calcium channel blockers, were found to remarkably inhibit the metabolism of CsA with an inhibitory rate of over 80%. Nimodipine inhibits the activity of CsA in rat liver microsomes with an IC_50_ of 20.54 ± 0.93 μM, while nisoldipine has an IC_50_ of 16.16 ± 0.78 μM. In *in vivo*, three groups of Sprague–Dawley rats were administered CsA with or without nimodipine or nisoldipine; the AUC_(0-t)_ and AUC_(0-∞)_ of CsA were significantly increased in the nimodipine group but not obviously in the nisoldipine group. Mechanistically, the inhibition mode of nimodipine on cyclosporine metabolism is a mixed inhibition. Our data show that gene polymorphisms of CYP3A4 and nimodipine remarkably affect the metabolism of CsA, thus providing a reference for the precise administration of CsA.

## Highlights

The kinetic parameters of cyclosporine metabolism by CYP3A4 variants are determined.
Nimodipine can inhibit the metabolism of cyclosporine.
Nimodipine is a mixed inhibitor of CYP3A4.

## Introduction

Cyclosporine (CsA) is a widely used immunosuppressant agent often prescribed for the prevention of allograft rejection after transplants and the treatment of rheumatoid arthritis, psoriasis, and other conditions. However, CsA has a narrow therapeutic range and high pharmacokinetic variability. Blood concentrations of CsA have been shown to exhibit high inter- and intra-individual variability (Zhang et al., 2013; Cvetkovic et al., 2017). Overexposure to CsA may result in adverse effects, such as viral infections, hepatotoxicity, nephrotoxicity, and post-transplant diabetes, while underexposure puts patients at risk of allograft rejection (Contreras-Castillo et al., 2021). Consequently, it is of vital importance to optimize the CsA dosing regimen based on the influence of gene polymorphisms on its metabolism.

CsA is mainly metabolized by CYP3A4 and CYP3A5. CYP3A4 has a more dominant role than CYP3A5 in the metabolism of CsA and primarily produces the metabolite AM1 (Li et al., 2019; Contreras-Castillo et al., 2021). The gene encoding CYP3A4 contains series of remarkable polymorphisms (Zanger and Schwab, 2013). In previous studies, our group discovered seven novel variants in the Han Chinese population, which were designated as CYP3A4*28–*34 (Hu et al., 2017). However, the impact of these CYP3A4 polymorphisms on CsA disposition remains unknown.

Drug–drug interactions (DDIs) are another factor influencing the blood levels of CsA. Interactions between CsA and HIV-1 protease inhibitors, such as lopinavir and ritonavir, and antifungal drugs, such as voriconazole and isoconazole, have been reported (Groll et al., 2004; Groll et al., 2017; Nicolas et al., 2017). Given that nimodipine is a commonly used cardiovascular drug, it is common for cardiovascular patients with rheumatoid arthritis and other autoimmune diseases to be co-administered nimodipine with CsA (He et al., 2014; Macdonald and Schweizer, 2017). Therefore, nimodipine was used in combination with CsA to study its influence on the metabolism of CYP3A4 on CsA both *in vivo* and *in vitro*.

In the present study, we investigated the metabolism of CsA from the view of pharmacogenetics and DDIs. The data produced here can promote the individual application of CsA.

## Methods

### Chemicals and materials

Cyclosporin A (CsA) was purchased from Shanghai Macklin Biochemical Co., Ltd. Its metabolite CsA M-17 (AM-1) was purchased from Toronto Research Chemicals Inc. Diazepam (internal standard, IS) was purchased from Shanghai Hanxiang Biological Technology Co., Ltd. Reduced nicotinamide adenine dinucleotide phosphate (NADPH) was obtained from Sigma-Aldrich Company (St Louis, MO). High-performance liquid chromatography (HPLC)-grade organic solvents were obtained from Merck (Darmstadt, Germany). Recombinant human CYP3A4 and cytochrome b5 were prepared by our group as described previously (Zhou et al., 2019). The rat liver microsomes (RLMs) and human liver microsomes (HLMs) were from Corning Life Sciences Co., Ltd. The information on 132 drugs is presented in Supplementary Table S1.

### Ultra-performance liquid chromatography–tandem mass spectrometry (UPLC–MS/MS) and operational conditions

The concentrations of CsA, its main metabolite AM1, and diazepam (IS) were detected by UPLC–MS/MS equipped with a triple quadrupole and electrospray ionization (ESI) source. This method has been referred to previously in the literature (Tszyrsznic et al., 2013; Rigo-Bonnin et al., 2015). Briefly, the analytes were separated on a UPLC BEH C18 column (2.1 mm × 50 mm, 1.7 μm particle size). The multiple reaction monitoring transitions were m/z 1202.9 → 1184.7 for CsA, m/z 1219 → 1200.2 for AM-1, and m/z 285 → 154 for diazepam. Solvent A (0.1% formic acid) and solvent B (acetonitrile) were applied as the mobile phase, with a gradient elution at a flow rate of 0.4 ml/min and an injection volume of 4 μl. The following stepwise gradient elution program was used: 90% A (0–0.5 min); 90%–10% A (0.5–1.0 min); 10% A (1.0–2.0 min); 10%–90% A (2–2.1 min); and 90% A (2.1–3.0 min).

### Recombinant human CYP3A4 enzymatic incubation assay

Human CYP3A4 and variants were expressed by a baculovirus expression system as previously described. The cell microsomes were prepared and determined by CO differential quantification (Zhou et al., 2019). The reaction system contained 1 pmol CYP3A4.1 or another CYP3A4 protein variant, 10 μg of cytochrome b5, 100 mM Tris-HCl buffer (pH 7.4), and 1–50 μM CsA at a final volume of 200 μl. Reduced nicotinamide adenine dinucleotide phosphate (NADPH) (1 mmol/L) was added into the mixture after preincubation at 37°C for 5 min to initiate the reaction. The mixture was incubated for another 40 min, then cooled at −80°C to suspend the reaction. Then, 400 μl of acetonitrile and 20 μl of diazepam (200 ng/ml) were added to the frozen mixture. The mixtures were vortexed for 2 min and centrifuged at 13,000 rpm for 10 min. Finally, 4 μl of the mixture was injected for UPLC-MS/MS analysis.

### *In vitro* kinetics study

A 200 μl system was incubated with either RLMs or HLMs (at a concentration of 0.15 mg/ml) containing drugs, CsA, RLMs or HLMs (all at a concentration of 0.15 mg/ml), Tris-HCl buffer, and 10 μl of NADPH (1 mM). To determine the IC_50_, nimodipine or nisoldipine was dissolved in methanol and diluted to 0.01, 0.1, 1, 10, 25, 50, and 100 μM, while the concentration of CsA was 20 μM (RLMs) or 2 μM (HLMs), close to the Km. To explore the inhibition mechanism of nimodipine on CsA in the HLM incubation system, the concentration of CsA was prepared at 0.5, 1, 2, and 4 μM according to the Km value, and the concentration of inhibitor was prepared at 0, 3, 6, and 12 μM according to the IC_50_ value. The rest of the operation was the same as already described.

### Animal experiments

Sprague–Dawley male rats weighing 200–250 g were purchased from Shanghai Laboratory Animal Center (SLAC). After 2 weeks of laboratory adaptation, the rats were randomly divided into three groups with six rats in each group. They were fasted for 12 h before the experiment and administered with bioequivalent dose for CsA, nimodipine, and nisoldipine (Lu et al., 2004; Zhang et al., 2013). Group A was given 40 mg/kg CsA, and groups B and C were given 24 mg/kg nimodipine and 2 mg/kg nisoldipine before the administration of CsA, respectively. All drugs were orally administered. At 0.5, 1, 2, 3, 4, 5, 6, 8, 12, 24, and 48 h after administration, blood samples were collected from the tail vein of rats into heparinized Eppendorf tubes. The collected blood samples were centrifuged to obtain the plasma. Then, 150 μl of acetonitrile and 20 μl of diazepam (IS; 100 ng/ml) were added to 50 μl of plasma, mixed and vortexed for 2 min, centrifuged for 10 min, and 4 μl of the supernatant was used for UPLC-MS/MS injection analysis.

### Statistical analysis

The enzyme kinetic parameters (Km and Vmax) were calculated by non-linear regression curve fitting using the computer program Prism version 6 (GraphPad Software Inc., San Diego, CA, United states). The mean concentration–time curve was drawn using Origin 8.0. The pharmacokinetic parameters were evaluated by DAS 3.0. All the statistical results were expressed as the average ± standard deviation (mean ± SD) and analyzed by SPSS 24.0 (*p* < 0.05 statistical significance).

## Results

### Kinetic profile of CYP3A4 and its variants on metabolizing CSA

**FIGURE 1:**
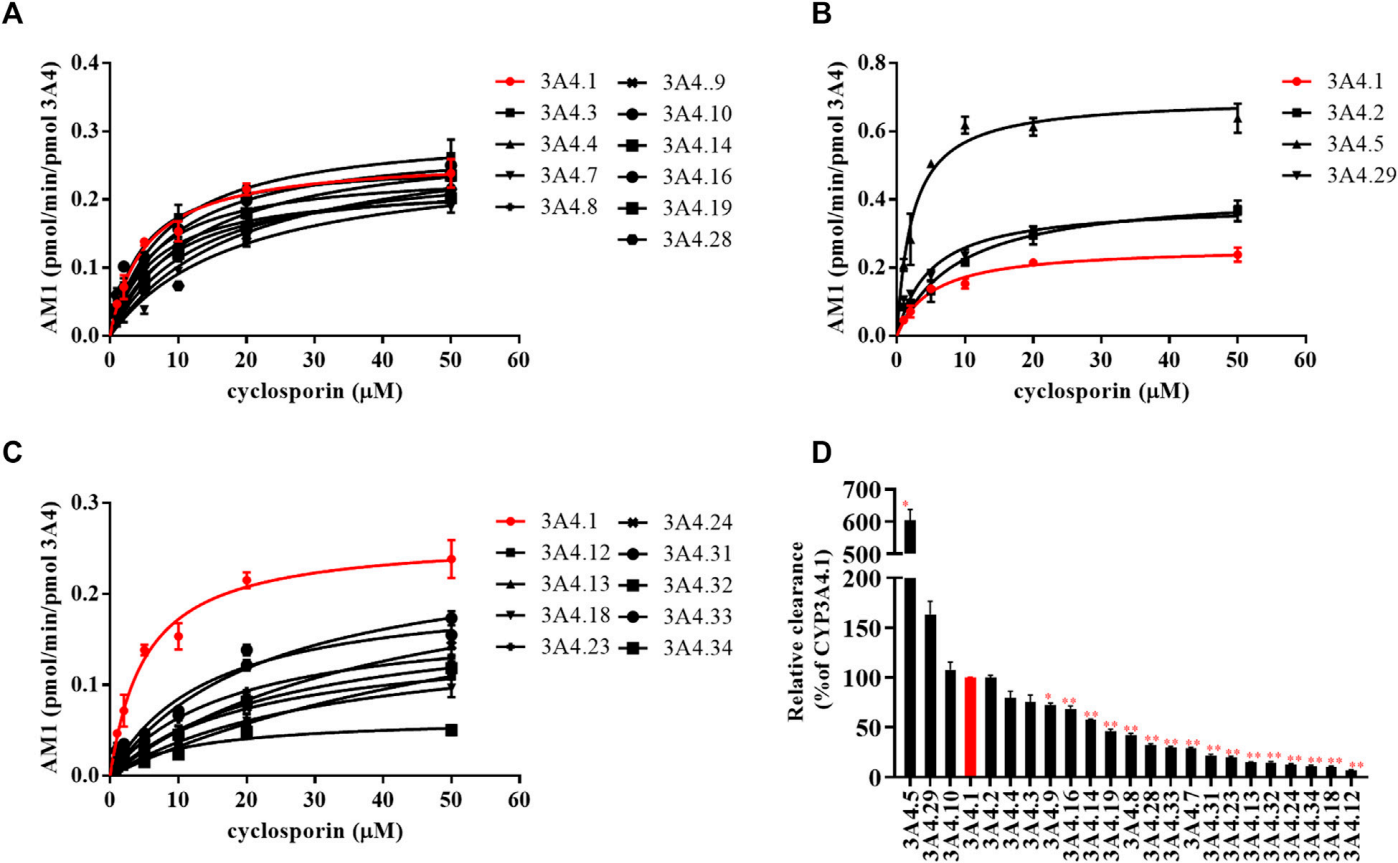
Michaelis–Menten curves of the enzymatic activities of CYP3A4.1 and other CYP3A4 variants on the metabolism of cyclosporin. **(A–C)** Relative clearance by CYP3A4 variants based on the metabolism of cyclosporin compared with CYP3A4.1 **(D)**. Data are presented as the means ± SD, *n* = 3. *p < 0.05, **p < 0.01.

**FIGURE 2:**
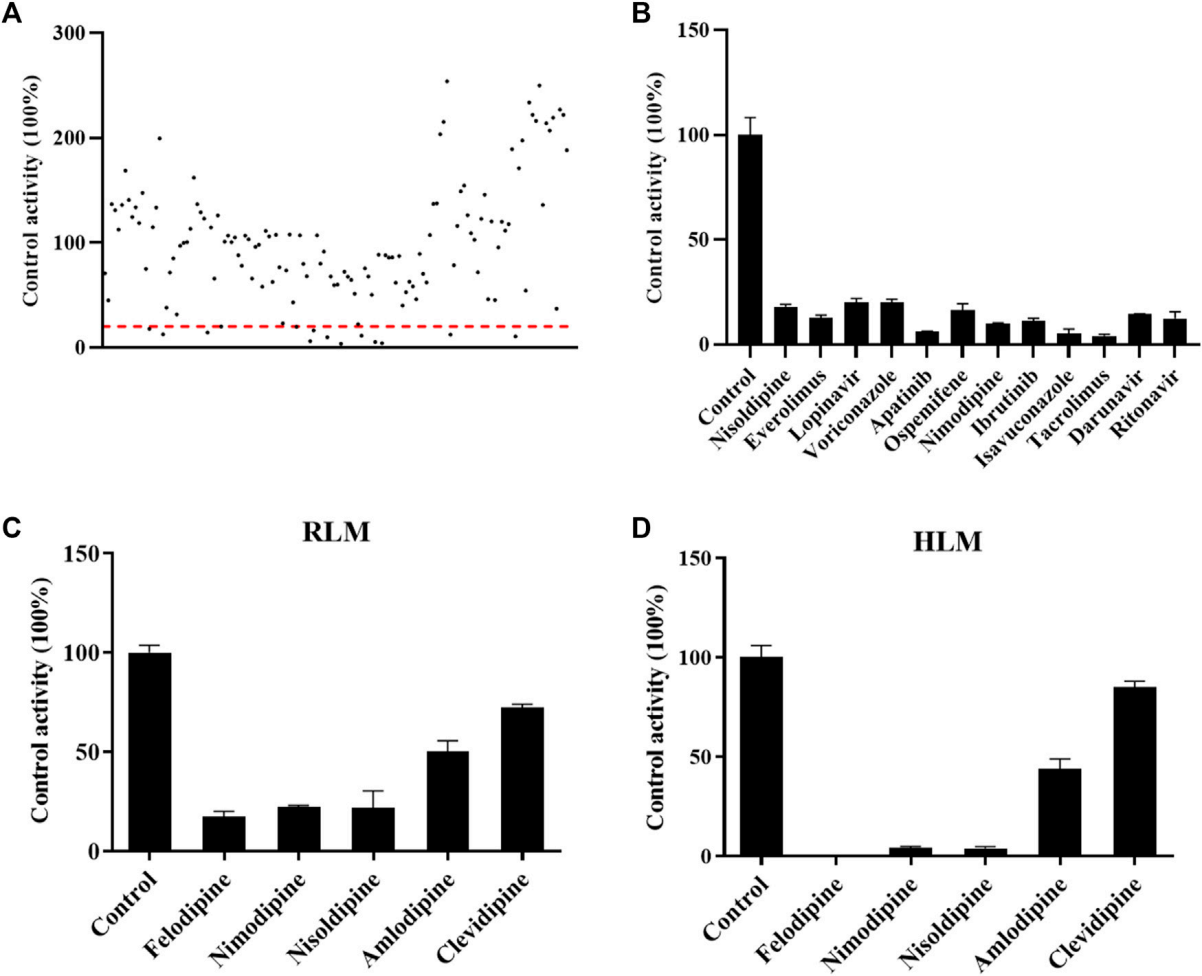
Drugs screened for inhibition of the metabolism of cyclosporin. The RLM incubation assay was performed as indicated in the method. **(A)** All drugs that were screened in RLM. **(B)** Drugs with an inhibition rate >80% in RLM. **(C)** and **(D)** Comparisons of the inhibitory effect of five antihypertension drugs on the metabolism of cyclosporine in RLMs and HLMs, respectively. Data are presented as the means ± SD.

The Michaelis–Menten curves of CsA were plotted after enzymatic incubation assays were performed. As shown in Figures 1A–C, the curves could be divided into three groups. In addition, the corresponding kinetic parameters are summarized in Table 1. The intrinsic clearance (Clint) value of CYP3A4.5 was 0.304 ± 0.017 μl/min/pmol and was about six times that of CYP3A4.1 (0.050 ± 0.002 μl/min/pmol). The relative clearance of CYP3A4.5 was remarkably higher than that of CYP3A4.1, suggesting significantly enhanced enzyme activity (Figure 2D). In contrast, AM1 was not detected in CYP3A4.20 due to weak enzymatic activity or lower concentrations exceeding the limit of detection. Clint values of 16 other variants (CYP3A4.7, 8, 9, 12, 13, 14, 16, 18, 19, 23, 24, 28, 31, 32, 33, and 34) ranged from 0.004 ± 0.000 to 0.034 ± 0.001 μl/min/pmol and the corresponding relative clearance rates were all distinctly lower than CYP3A4.1, indicating that these variants exhibit a significantly poor metabolic capacity for CsA. Clint values of CYP3A4.2, 3, 4, 10, and 29 ranged from 0.038 ± 0.003 to 0.078 ± 0.006 μl/min/pmol, which were close to the Clint of CYP3A4.1 (0.050 ± 0.002 μl/min/pmol) (Table 1).

**TABLE 1:** Kinetic parameters for AM1 activity of CYP3A4.1 and 23 other CYP3A4 variants on cyclosporin metabolism.

Variant	V_max_ (pmol/min/pmol P450)	K_m_ (μM)	Clint (V_max_/K_m_) (μl/min/pmol P450)
3A4.1	.262 ± .016	5.204 ± .494	.050 ± .002
3A4.2	.427 ± .016**	8.958 ± .498*	.048 ± .001
3A4.3	.303 ± .024	8.018 ± .604	.038 ± .004
3A4.4	.244 ± .007	6.450 ± .547	.038 ± .003
3A4.5	.698 ± .021***	2.296 ± .082	.304 ± .017*
3A4.7	.264 ± .005	19.030 ± .823**	.014 ± .001**
3A4.8	.261 ± .008	13.093 ± .947*	.020 ± .001***
3A4.9	.222 ± .007	6.436 ± .258	.034 ± .001**
3A4.10	.256 ± .005	4.730 ± .408	.054 ± .004
3A4.12	.277 ± .018	76.760 ± 8.151*	.004 ± .000**
3A4.13	.152 ± .006*	21.140 ± .296***	.007 ± .000**
3A4.14	.231 ± .004	8.384 ± .235*	.028 ± .000**
3A4.16	.286 ± .010	8.717 ± .672	.033 ± .001**
3A4.18	.163 ± .042	33.600 ± 12.046	.005 ± .001***
3A4.19	.292 ± .022	12.570 ± .696**	.023 ± .001**
3A4.20	N.D	N.D	N.D
3A4.23	0.175 ± 0.005	17.543 ± 1.830	.010 ± .001***
3A4.24	0.261 ± 0.016	42.963 ± 0.751***	.006 ± .000**
3A4.28	0.295 ± 0.034	18.953 ± 1.496*	.016 ± .001**
3A4.29	0.387 ± 0.016*	4.966 ± .407	.078 ± .006
3A4.31	0.253 ± 0.014	22.893 ± 1.643*	.011 ± .001***
3A4.32	0.179 ± 0.012	25.490 ± .849***	.007 ± .000**
3A4.33	0.206 ± 0.022	14.400 ± 1.609	.014 ± .001**
3A4.34	0.064 ± 0.008**	11.660 ± 1.654	.006 ± .000**

Significantly different from wild-type CYP3A4, *p < .05, **p < .01, ***p < .001. ND, not determined.

### Identification of the drugs that inhibit the metabolism of CsA

As shown in Figure 2A, the effect of 132 drugs on suppressing the activity of CsA was determined. A total of 12 drugs were identified with an inhibitory rate greater than 80%, namely, nisoldipine, everolimus, lopinavir, voriconazole, apatinib, ospemifene, nimodipine, ibrutinib, isavuconazole, tacrolimus, darunavir, and ritonavir (Figure 2B). Among these drugs, calcium channel blockers caught our attention. Therefore, five representative drugs were analyzed both in RLMs and HLMs. As shown in Figures 2C, D, felodipine, nimodipine, and nisoldipine almost completely blocked the metabolism of CsA.

### Nimodipine and nisoldipine inhibit the disposition of CsA both *in vitro* and *in vivo*

**FIGURE 3:**
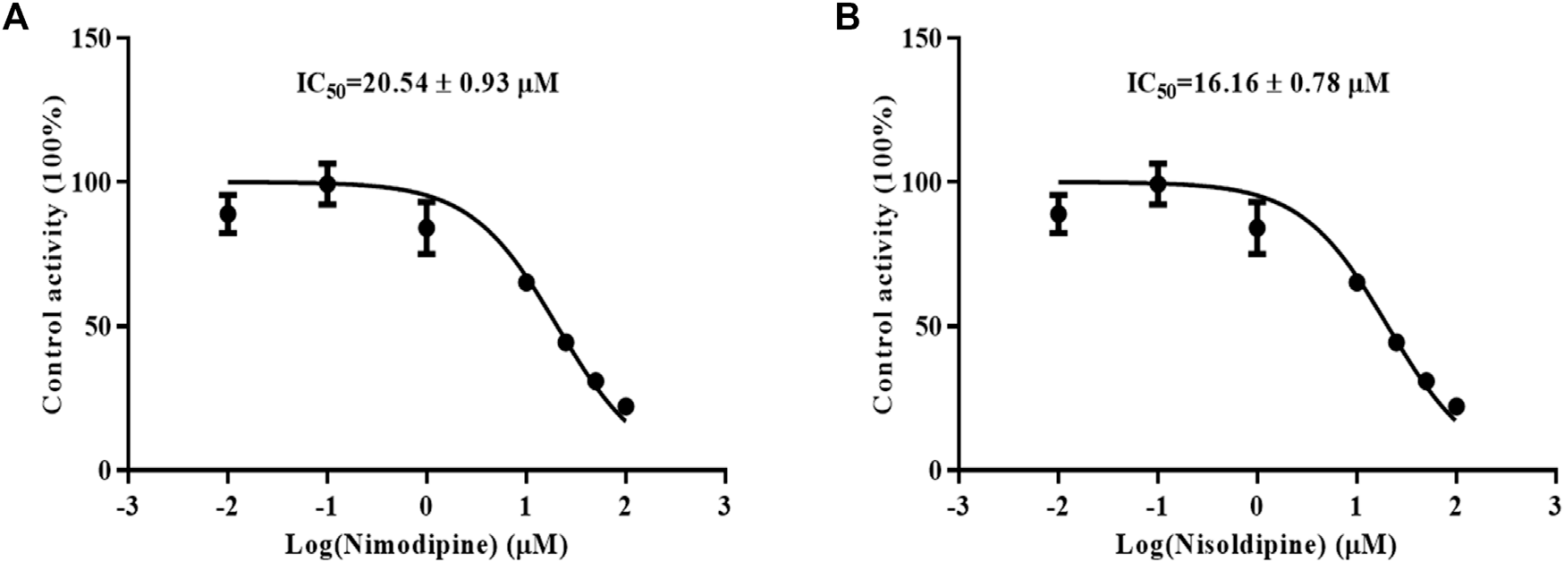
Effects of nimodipine **(A)** and nisoldipine **(B)** on the metabolism of cyclosporine in RLMs. Data are presented as the means ± SD, *n* = 3.

Because the influence of felodipine on CsA has been reported, we evaluated the efficiency of nimodipine and nisoldipine in inhibiting CsA metabolism using RLMs. The IC_50_ values were determined, which were 20.54 ± 0.93 μM for nimodipine and 16.16 ± 0.78 μM for nisoldipine (Figures 3A,B).

**FIGURE 4:**
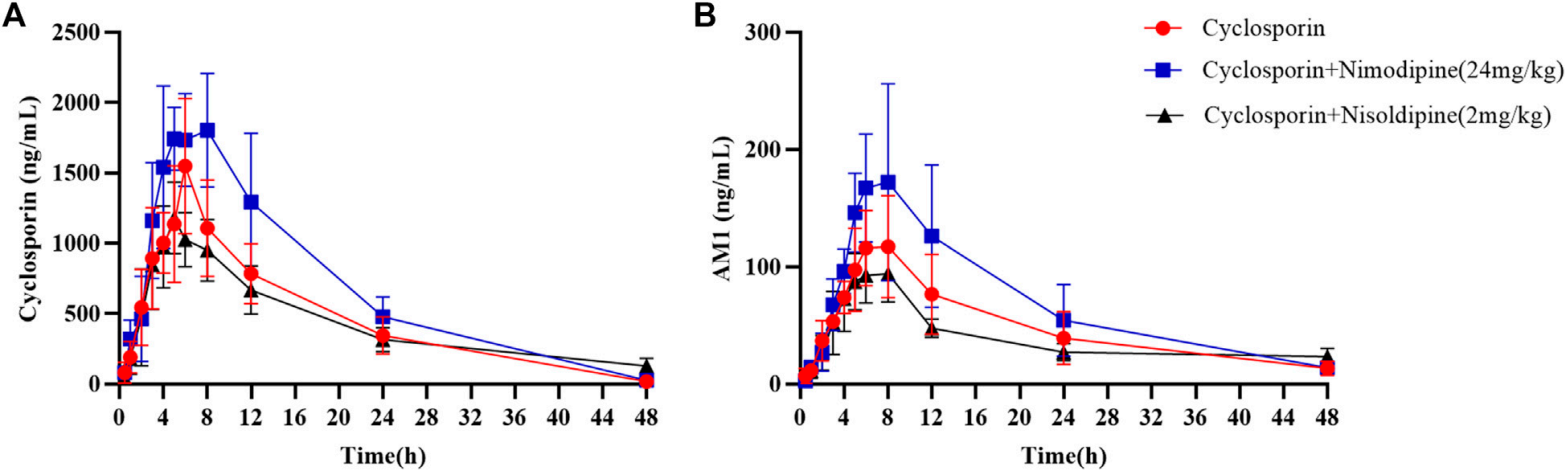
Mean concentration–time curve of cyclosporine **(A)** and its metabolite AM1 **(B)** in the three groups. Data are presented as the means ± SD, *n* = 6.

An *in vivo* experiment was also performed. Rats were administered CsA with or without nimodipine or nisoldipine. As shown in Figure 4A, the mean plasma concentration time curves of CsA increased significantly when co-administered with nimodipine. The area under the curve AUC_(0-t)_ and AUC_(0-∞)_ of CsA significantly increased and the apparent blood clearance (CL_z/F_) significantly decreased (32,075.93 ± 8,210.72 and 32,337.21 ± 8,156.34 μg/L·h, and 1.33 ± 0.46 L/h/kg, respectively) (Table 2). However, the main pharmacokinetic parameters of AM1 showed no significant differences (Figure 4B; Tables 2, 3). In contrast, with a combination of CsA and nisoldipine, the elimination half time (t_1/2z_) and apparent volume of distribution (V_z/F_) of CsA significantly increased (13.04 ± 2.56 h and 34.82 ± 6.79 L/kg, respectively). Meanwhile, the AUC _(0-t)_ of AM1 in this group remarkably decreased and the V_z/F_ of AM1 remarkably increased (1,548.46 ± 482.43 μg/L·h and 494.23 ± 201.15 L/kg, respectively) (Figure 4; Tables 2, 3).

**TABLE 2:** Main pharmacokinetics parameters of cyclosporin in three groups of rats (*n* = 6).

Parameter	Cyclosporin (40 mg/kg)	Cyclosporin (40 mg/kg) + nimodipine (24 mg/kg)	Cyclosporin (40 mg/kg) + nisoldipine (2 mg/kg)
AUC_(0-t)_ (μg/L·h)	19,032.20 ± 7,160.00	32,075.93 ± 8,210.72*	18,919.37 ± 5,668.04
AUC_(0-∞)_ (μg/L·h)	22,523.78 ± 2,530.32	32,337.21 ± 8,156.34*	22,224.93 ± 6,085.61
t_1/2z_ (h)	7.97 ± 3.24	6.62 ± 0.94	13.04 ± 2.56*
T_max_ (h)	6.00 ± 1.10	5.67 ± 1.86	5.00 ± 1.67
V_z/F_ (L/kg)	20.37 ± 6.95	13.11 ± 6.54	34.82 ± 6.79**
CL_z/F_ (L/h/kg)	1.79 ± 0.19	1.33 ± 0.46*	1.91 ± 0.48
C_max_ (μg/L)	1,690.51 ± 373.77	1,933.95 ± 382.31	1,244.62 ± 194.62

AUC, area under the blood concentration–time curve; t_1/2z_, elimination half time; T_max_, peak time; V_z/F_, apparent volume of distribution; CL_z/F_, blood clearance; C_max_, maximum blood concentration.

*p < 0.05, in comparison with the control group.

**TABLE 3:** Main pharmacokinetics parameters of AM1 in three groups of rats (*n* = 6).

Parameter	Cyclosporin (40 mg/kg)	Cyclosporin (40 mg/kg) + nimodipine (24 mg/kg)	Cyclosporin (40 mg/kg) + nisoldipine (2 mg/kg)
AUC_(0-t)_ (μg/L·h)	2,275.37 ± 620.96	3,291.60 ± 1,213.46	1,548.46 ± 482.43*
AUC_(0-∞)_ (μg/L·h)	2,542.74 ± 779.21	3,504.89 ± 1,223.40	2,128.73 ± 887.82
t_1/2z_ (h)	12.10 ± 9.00	10.83 ± 3.52	19.58 ± 13.45
T_max_ (h)	6.83 ± 1.33	7.00 ± 2.68	6.17 ± 1.60
V_z/F_ (L/kg)	264.78 ± 145.01	198.16 ± 99.97	494.23 ± 201.15*
CL_z/F_ (L/h/kg)	17.03 ± 5.31	12.42 ± 3.60	21.26 ± 7.31
C_max_ (μg/L)	141.21 ± 30.64	192.68 ± 69.42	110.32 ± 19.52

AUC, area under the blood concentration–time curve; t_1/2z_, elimination half time; T_max_, peak time; V_z/F_, apparent volume of distribution; CL_z/F_, blood clearance; C_max_, maximum blood concentration.

### Nimodipine inhibits the metabolism of CsA in a mixed mode

**FIGURE 5:**
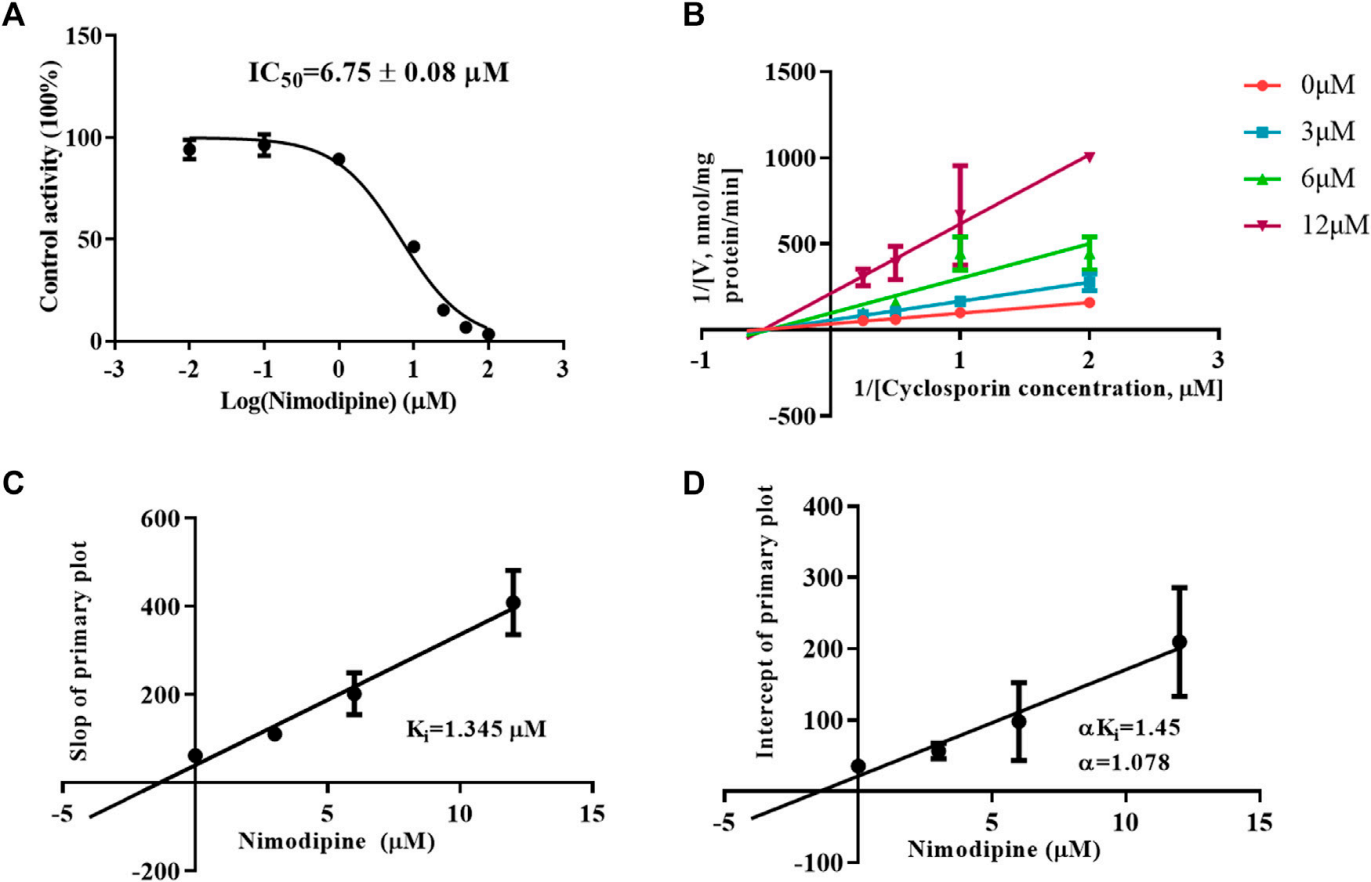
Mechanism of nimodipine on cyclosporine metabolism in HLMs. **(A)** Nimodipine at various concentrations affected the metabolism of cyclosporine, and the half-maximal inhibitory concentration (IC_50_) in HLMs was determined. Data are presented as the means ± SD, *n* = 3. **(B–D)** Primary Lineweaver–Burk plot and a secondary plot for Ki and αKi in the inhibition of cyclosporin metabolism by nimodipine at various concentrations in HLMs. Data are presented as the mean ± SD, *n* = 3.

To investigate the inhibitory mechanism, a kinetic study was carried out using HLMs. As shown in Figure 5A, nimodipine potently inhibited the metabolism of CsA with an IC_50_ of 6.75 ± 0.08 μM. The Lineweaver Burk plot shows that the straight lines are close to intersect at a point on X-axis, while the further slope diagram and y-intercept diagram show that the Ki value was 1.345 μM and the α value was 1.078 (α ≠ 1). Therefore, the collective data indicated that this inhibitory effect followed the mixed mechanism (Figures 5B–D).

## Discussion

CsA has a narrow therapeutic window and displays broad pharmacokinetic variability (Cossart et al., 2021). CYP3A4 is the main metabolic pathway involved in the disposition of CsA. CsA shows genetic polymorphisms and can be induced and inhibited by other exogenous substances (Hu et al., 2017; Contreras-Castillo et al., 2021). All these situations potentially lead to changes in the dose–response relationship of CsA and diverse clinical outcomes. Although CYP3A4 genetic polymorphisms have been investigated before, few studies have evaluated the effects of CYP3A4 genetic polymorphisms on the pharmacokinetics of CsA.

Our work used CYP3A4.1 as a reference to assess the enzyme kinetic parameters of 23 other CYP3A4 variants on CsA metabolism *in vitro*. Depending on the relative clearance rates of CsA, we found that CYP3A4.5 and 29 had high metabolic activity, which is relatively consistent with previously published findings (Han et al., 2021a; Han et al., 2021b). As a rapidly metabolized enzyme, our data show that the relative clearance of CYP3A4.5 (603.52%) was markedly higher than that of CYP3A4.29 (163.45%), which is different from Han et al.’s finding that the relative clearance of CYP3A4.29 *in vitro* was approximately 2.8–3.6 times that of CYP3A4.5. The underlying cause may be that there are wide differences in the structure of the substrates, which bind to different binding domains on CYP3A4 variants, showing metabolic differences of the same CYP3A4 variants (Zhou, 2008; Sevrioukova, 2019; Han et al., 2021b). Because the frequency distribution of the CYP3A4*5 allele is 0.5% in Han Chinese and 0.7% in Taiwan Chinese (Zhou et al., 2011; Werk and Cascorbi, 2014; Hu et al., 2017) and the population base is large, these CYP3A4*5 carriers may experience poor therapeutic effects when treated with standard therapeutic doses of CsA.

Additionally, 16 variants (CYP3A4.7–9, 12–14, 16, 18, 19, 23, 24, 28, and 31–34) showed low catalytic activities, ranging from 7.19% to 72.10%, compared with CYP3A4.1. The variants CYP3A4.14, 16, 18, 19, 28, and 31–34 are mainly distributed in Asian and African populations (Zhou et al., 2011; Werk and Cascorbi, 2014; Hu et al., 2017). Compared with wild-type carriers, patients with these CYP3A4 variants and treated with CsA show lower drug metabolic turnover. AM1 was not detected in CYP3A4.20, which has been reported to be unable to integrate into heme, and has been considered to be non-functional and to have no catalytic activity for CsA (Westlind-Johnsson et al., 2006).

In previous studies, it has been determined that there are mutations in exon 5 of the CYP3A4.8 allele (Eiselt et al., 2001). CYP3A4.12 (21905C > T) in exon 11 results in an L373F change in the CYP3A4 protein (Lamba et al., 2002); the amino acid Leu in CYP3A4.13 replaces Pro 416; CYP3A4.14 (T44C) in exon 1 results in an L15P change; CYP3A4.16 (15612C > G ) in exon 7 results in a T185S amino acid substitution; and CYP3A4.17 produces an amino acid substitution of F189S on exon 7 (Lamba et al., 2002; Kang et al., 2009; Han et al., 2021b). These changes disrupt the structure and function of the protein, leading to decreased catalytic activity compared with the wild-type enzyme.

The remaining CYP3A4.2, 3, 4, 10, and 29 variants displayed similar relative intrinsic clearance values for CsA compared with CYP3A4.1. Thus, these findings aid in the establishment of a direct relationship between gene function and metabolic phenotype.

Enzymatic activity can be affected not only by genetic polymorphisms but also by interactions with drugs (Yu et al., 2018). Our studies *in vitro* found that nimodipine was an inhibitor of CsA, and its IC_50_ values were 20.54 ± 0.93 μM in RLMs and 6.75 ± 0.08 μM in HLMs, as shown in Figures 3A, 5A. Nimodipine’s inhibitory ability in HLMs was about three times that in RLMs, showing the obvious species difference.

Our results reveal that not only do nimodipine and nisoldipine have a significant inhibitory effect on CYP3A4 *in vitro*, they are also inhibitory *in vivo* in rats. The average plasma concentration–time curves of CsA and its metabolite AM1 and their corresponding pharmacokinetic characteristics changed clearly after adding nimodipine, but this was not significant in the nisoldipine combo group. Compared with the CsA-only group, the AUC_(0-t)_ and AUC_(0-∞)_ of CsA were both increased by 68.54% and 43.57%, respectively, and the CL_z/F_ of CsA was decreased by 25.70% in the nimodipine coadministration groups. Interestingly, the AUC_(0-t)_ and AUC_(0-∞)_ of AM1 slightly increased in the nimodipine group, which may be due to the reduction of the first-pass effect on CsA with the drug combination and the increased bioavailability of its metabolite AM1. To further explore the inhibitory effect of nimodipine on CsA, we studied its inhibitory mechanism in HLMs. The results indicate that the inhibitory effect of nimodipine on CsA is a mixed inhibition.

No prior studies have assessed the interaction of CsA and nimodipine. Our results indicate that the co-administration of CsA and nimodipine may lead to higher exposure to CsA, and dosage adjustments of CsA may be needed. Previous studies have indicated that the metabolites of CsA have lower immunosuppressive activity than the parent drug (Christians and Sewing, 1993; Fahr, 1993). High CsA concentrations exert a stronger immunosuppressive effect, but may not improve the disease, and even induce infection and aggravate the condition (Zamora-Legoff et al., 2016; Turshudzhyan, 2021). In addition, excessive CsA exposure may increase CsA-induced neurotoxicity, including headaches, disorientation, seizures, visual disturbance, hepatotoxicity, hypertension, and gingival swelling (Sikora-Wiorkowska et al., 2019; Teshome et al., 2020; Danish et al., 2021).

Our work reveals the relationship between the kinetic characteristics of CYP3A4 and its variants and provides basic data for metabolic characterizations of combinations of CsA and nimodipine. Prompt recognition of individual genotypes and characteristics of interaction and early intervention can improve the efficacy of CsA and reduce the adverse reaction of potential DDIs.

## Conclusion

In this study, the kinetic characteristics of CsA varied by different CYP3A4 variants. Nimodipine clearly inhibited the metabolism of CsA both *in vivo* and *in vitro*. The collective data demonstrate that the DDIs and CYP3A4 polymorphisms can influence the pharmacokinetic profile of CsA. These results provide a basic data set for individualized treatment with CsA.

## Data Availability

The raw data supporting the conclusion of this article will be made available by the authors, without undue reservation.

## Abbreviations

Clint, intrinsic clearance; CsA, cyclosporine; CYP3A4, cytochrome P450 3A4; DDI, drug–drug interaction; HPLC, high-performance liquid chromatography; Km, Michaelis–Menten constant; NADPH, reduced nicotinamide adenine dinucleotide phosphate; ND, not determined; UPLC-MS/MS, ultra-performance liquid chromatography–tandem mass spectrometry; Vmax, maximum enzymatic activity.

## References

[B1] Christians U. Sewing K. F. (1993). Cyclosporin metabolism in transplant patients. Pharmacol. Ther. 57, 291–345. 10.1016/0163-7258(93)90059-m 8361996

[B2] Contreras-Castillo S. Plaza A. Stojanova J. Navarro G. Carmona R. Corvalan F. (2021). Effect of CYP3A4, CYP3A5, MDR1 and POR genetic polymorphisms in immunosuppressive treatment in Chilean kidney transplanted patients. Front. Pharmacol. 12, 674117. 10.3389/fphar.2021.674117 34938174 PMC8685429

[B3] Cossart A. R. Isbel N. M. Scuderi C. Campbell S. B. Staatz C. E. (2021). Pharmacokinetic and pharmacodynamic considerations in relation to calcineurin usage in elderly kidney transplant recipients. Front. Pharmacol. 12, 635165. 10.3389/fphar.2021.635165 33912051 PMC8072471

[B4] Cvetkovic M. Zivkovic M. Bundalo M. Gojkovic I. Spasojevic-Dimitrijeva B. Stankovic A. (2017). Effect of age and allele variants of CYP3A5, CYP3A4, and por genes on the pharmacokinetics of cyclosporin A in pediatric renal transplant recipients from Serbia. Ther. Drug Monit. 39, 589–595. 10.1097/FTD.0000000000000442 29135906

[B5] Danish A. Mughal S. I. Zaidi U. Dildar S. Samad S. Jamal A. (2021). Frequency and risk factors of cyclosporine-induced neurotoxicity in allogeneic stem cell transplant recipients. Cureus 13, e19824. 10.7759/cureus.19824 34963841 PMC8696087

[B6] Eiselt R. Domanski T. L. Zibat A. Mueller R. Presecan-Siedel E. Hustert E. (2001). Identification and functional characterization of eight CYP3A4 protein variants. Pharmacogenetics 11, 447–458. 10.1097/00008571-200107000-00008 11470997

[B7] Fahr A. (1993). Cyclosporin clinical pharmacokinetics. Clin. Pharmacokinet. 24, 472–495. 10.2165/00003088-199324060-00004 8513650

[B8] Groll A. H. Desai A. Han D. Howieson C. Kato K. Akhtar S. (2017). Pharmacokinetic assessment of drug-drug interactions of isavuconazole with the immunosuppressants cyclosporine, mycophenolic acid, prednisolone, sirolimus, and tacrolimus in healthy adults. Clin. Pharmacol. Drug Dev. 6, 76–85. 10.1002/cpdd.284 27273343 PMC5298005

[B9] Groll A. H. Kolve H. Ehlert K. Paulussen M. Vormoor J. (2004). Pharmacokinetic interaction between voriconazole and ciclosporin A following allogeneic bone marrow transplantation. J. Antimicrob. Chemother. 53, 113–114. 10.1093/jac/dkh022 14657088

[B10] Han M. Qian J. Ye Z. Xu R. Chen D. Xie S. (2021a). Functional assessment of the effects of CYP3A4 variants on acalabrutinib metabolism *in vitro* . Chem. Biol. Interact. 345, 109559. 10.1016/j.cbi.2021.109559 34153224

[B11] Han M. Zhang X. Ye Z. Wang J. Qian J. Hu G. (2021b). Functional evaluation of vandetanib metabolism by CYP3A4 variants and potential drug interactions *in vitro* . Chem. Biol. Interact. 350, 109700. 10.1016/j.cbi.2021.109700 34648813

[B12] He J. Zhang Y. H. Xu T. Zhao Q. Wang D. L. Chen C. S. (2014). Effects of immediate blood pressure reduction on death and major disability in patients with acute ischemic stroke the CATIS randomized clinical trial. Jama-Journal Am. Med. Assoc. 311, 479–489. 10.1001/jama.2013.282543 24240777

[B13] Hu G. X. Dai D. P. Wang H. Huang X. X. Zhou X. Y. Cai J. (2017). Systematic screening for CYP3A4 genetic polymorphisms in a Han Chinese population. Pharmacogenomics 18, 369–379. 10.2217/pgs-2016-0179 28244811

[B14] Kang Y. S. Park S. Y. Yim C. H. Kwak H. S. Gajendrarao P. Krishnamoorthy N. (2009). The CYP3A4*18 genotype in the cytochrome P450 3A4 gene, a rapid metabolizer of sex steroids, is associated with low bone mineral density. Clin. Pharmacol. Ther. 85, 312–318. 10.1038/clpt.2008.215 19020497

[B15] Lamba J. K. Lin Y. S. Thummel K. Daly A. Watkins P. B. Strom S. (2002). Common allelic variants of cytochrome P4503A4 and their prevalence in different populations. Pharmacogenetics 12, 121–132. 10.1097/00008571-200203000-00006 11875366

[B16] Li T. F. Hu L. Ma X. L. Huang L. Liu X. M. Luo X. X. (2019). Population pharmacokinetics of cyclosporine in Chinese children receiving hematopoietic stem cell transplantation. Acta Pharmacol. Sin. 40, 1603–1610. 10.1038/s41401-019-0277-x 31341257 PMC7471407

[B17] Lu S. R. Liao Y. C. Fuh J. L. Lirng J. F. Wang S. J. (2004). Nimodipine for treatment of primary thunderclap headache. Neurology 62, 1414–1416. 10.1212/01.wnl.0000120669.85649.77 15111686

[B18] Macdonald R. L. Schweizer T. A. (2017). Spontaneous subarachnoid haemorrhage. Lancet 389, 655–666. 10.1016/S0140-6736(16)30668-7 27637674

[B19] Nicolas D. Ambrosioni J. Sued O. Brunet M. Lopez-Dieguez M. Manzardo C. (2017). Cyclosporine A in addition to standard art during primary HIV-1 infection: Pilot randomized clinical trial. J. Antimicrob. Chemother. 72, 829–836. 10.1093/jac/dkw462 27999018

[B20] Rigo-Bonnin R. Arbiol-Roca A. de Aledo-Castillo J. M. G. Alia P. (2015). Simultaneous measurement of cyclosporine A, everolimus, sirolimus and tacrolimus concentrations in human blood by UPLC-MS/MS. Chromatographia 78, 1459–1474. 10.1007/s10337-015-2981-0

[B21] Sevrioukova I. (2019). Interaction of human drug-metabolizing CYP3A4 with small inhibitory molecules. Biochemistry 58, 930–939. 10.1021/acs.biochem.8b01221 30676743 PMC6448587

[B22] Sikora-Wiorkowska A. Smolen A. Czechowska G. Wiorkowski K. Korolczuk A. (2019). The role of PPAR gamma agonists - rosiglitazone and 15-deoxy-Δ12, 14-prostaglandin J2 in experimental cyclosporine A hepatotoxicity. J. Physiol. Pharmacol. 70. 10.26402/jpp.2019.6.07 32084649

[B23] Teshome A. Girma B. Aniley Z. (2020). The efficacy of azithromycin on cyclosporine-induced gingival enlargement: Systematic review and meta-analysis. J. oral Biol. craniofacial Res. 10, 214–219. 10.1016/j.jobcr.2019.12.005 PMC7254476 32489824

[B24] Tszyrsznic W. Borowiec A. Pawlowska E. Jazwiec R. Zochowska D. Bartlomiejczyk I. (2013). Two rapid ultra performance liquid chromatography/tandem mass spectrometry (UPLC/MS/MS) methods with common sample pretreatment for therapeutic drug monitoring of immunosuppressants compared to immunoassay. J. Chromatogr. B 928, 9–15. 10.1016/j.jchromb.2013.03.014 23584041

[B25] Turshudzhyan A. (2021). Post-renal transplant malignancies: Opportunities for prevention and early screening. Cancer Treat. Res. Commun. 26, 100283. 10.1016/j.ctarc.2020.100283 33338850

[B26] Werk A. N. Cascorbi I. (2014). Functional gene variants of CYP3A4. Clin. Pharmacol. Ther. 96, 340–348. 10.1038/clpt.2014.129 24926778

[B27] Westlind-Johnsson A. Hermann R. Huennemeyer A. Hauns B. Lahu G. Nassr N. (2006). Identification and characterization of CYP3A4*20, a novel rare CYP3A4 allele without functional activity. Clin. Pharmacol. Ther. 79, 339–349. 10.1016/j.clpt.2005.11.015 16580902

[B28] Yu C. P. Huang C. Y. Lin S. P. Hou Y. C. (2018). Activation of P-glycoprotein and CYP 3A by Coptidis Rhizoma *in vivo*: Using cyclosporine as a probe substrate in rats. J. Food Drug Anal. 26, S125–S132. 10.1016/j.jfda.2017.11.005 29703381 PMC9326880

[B29] Zamora-Legoff J. A. Krause M. L. Crowson C. S. Ryu J. H. Matteson E. L. (2016). Risk of serious infection in patients with rheumatoid arthritis-associated interstitial lung disease. Clin. Rheumatol. 35, 2585–2589. 10.1007/s10067-016-3357-z 27448151

[B30] Zanger U. M. Schwab M. (2013). Cytochrome P450 enzymes in drug metabolism: Regulation of gene expression, enzyme activities, and impact of genetic variation. Pharmacol. Ther. 138, 103–141. 10.1016/j.pharmthera.2012.12.007 23333322

[B31] Zhang Y. Li J. L. Fu Q. Wang X. D. Liu L. S. Wang C. X. (2013). Associations of ABCB1, NFKB1, CYP3A, and NR1I2 polymorphisms with cyclosporine trough concentrations in Chinese renal transplant recipients. Acta Pharmacol. Sin. 34, 555–560. 10.1038/aps.2012.200 23503472 PMC4002791

[B32] Zhou Q. Yu X. Shu C. Cai Y. Gong W. Wang X. (2011). Analysis of CYP3A4 genetic polymorphisms in Han Chinese. J. Hum. Genet. 56, 415–422. 10.1038/jhg.2011.30 21412247

[B33] Zhou S. F. (2008). Drugs behave as substrates, inhibitors and inducers of human cytochrome P450 3A4. Curr. Drug Metab. 9, 310–322. 10.2174/138920008784220664 18473749

[B34] Zhou X. Y. Hu X. X. Wang C. C. Lu X. R. Chen Z. Liu Q. (2019). Enzymatic activities of CYP3A4 allelic variants on quinine 3-hydroxylation *in vitro* . Front. Pharmacol. 10, 591. 10.3389/fphar.2019.00591 31214030 PMC6555127

