# Supplementary material for: Functional evaluation of cyclosporine metabolism by CYP3A4 variants and potential drug interactions
Source: Front Pharmacol. 2023 Jan 6;13:1044817. doi: 10.3389/fphar.2022.1044817 (PMC9852833; doi:10.3389/fphar.2022.1044817)
Supplement: Supplementary file 1 [file Table1.DOCX]

**Supplementary Table S1.** The information about the 132 drugs.

| Name | CAS | company |
| --- | --- | --- |
| Loperamide hydrochloride | 34552-83-5 | Shanghai Canspec Scientific Instruments Co., Ltd |
| Lovastatin | 75330-75-5 | Shanghai Canspec Scientific Instruments Co., Ltd |
| Rifampicin | 13292-46-1 | Shanghai Canspec Scientific Instruments Co., Ltd |
| Rosuvastatin calcium | 147098-20-2 | Shanghai Canspec Scientific Instruments Co., Ltd |
| Sorafenib tosylate | 475207-59-1 | Shanghai Canspec Scientific Instruments Co., Ltd |
| Triptolide | 38748-32-2 | Shanghai Canspec Scientific Instruments Co., Ltd |
| Tamoxifen | 10540-29-1 | Shanghai Canspec Scientific Instruments Co., Ltd |
| Vortioxetine | 508233-74-7 | Beijing Sunflower and Technology Development Co., Ltd |
| Brivaracetam | 357336-20-0 | Shanghai Canspec Scientific Instruments Co., Ltd |
| Tropisetron | 89565-68-4 | Shanghai Canspec Scientific Instruments Co., Ltd |
| Hydrocortisone | 50-23-7 | Shanghai Canspec Scientific Instruments Co., Ltd |
| Sulfaphenazole | 526-08-9 | Shanghai Canspec Scientific Instruments Co., Ltd |
| Nootkatone | 4674-50-4 | Shanghai Canspec Scientific Instruments Co., Ltd |
| Nisoldipine | 63675-72-9 | Shanghai Canspec Scientific Instruments Co., Ltd |
| Quinine | 130-95-0 | Shanghai Canspec Scientific Instruments Co., Ltd |
| Irbesartan | 138402-11-6 | Shanghai Canspec Scientific Instruments Co., Ltd |
| Warfarin sodium | 129-06-6 | Shanghai Canspec Scientific Instruments Co., Ltd |
| Everolimus | 159351-69-6 | Shanghai Canspec Scientific Instruments Co., Ltd |
| Dronedarone hydrochloride | 141625-93-6 | Shanghai Canspec Scientific Instruments Co., Ltd |
| Duloxetine hydrochloride | 136434-34-9 | Shanghai Canspec Scientific Instruments Co., Ltd |
| Donepezil Hydrochloride | 120011-70-3 | Shanghai Canspec Scientific Instruments Co., Ltd |
| Furazolidone | 67-45-8 | Beijing Sunflower and Technology Development Co., Ltd |
| Dimenhydrinate | 523-87-5 | Shanghai Canspec Scientific Instruments Co., Ltd |
| Rosiglitazone maleate | 155141-29-0 | Shanghai Canspec Scientific Instruments Co., Ltd |
| Clomipramine hydrochloride | 17321-77-6 | Shanghai Canspec Scientific Instruments Co., Ltd |
| Lornoxicam | 70374-39-9 | Shanghai Canspec Scientific Instruments Co., Ltd |
| Lacosamide | 175481-36-4 | Shanghai Canspec Scientific Instruments Co., Ltd |
| Azilsartan | 147403-03-0 | Shanghai Canspec Scientific Instruments Co., Ltd |
| Acetazolamide | 59-66-5 | Shanghai Canspec Scientific Instruments Co., Ltd |
| Apixaban | 503612-47-3 | Shanghai Canspec Scientific Instruments Co., Ltd |
| Darunavir | 206361-99-1 | Shanghai Canspec Scientific Instruments Co., Ltd |
| Modafinil | 68693-11-8 | Shanghai Canspec Scientific Instruments Co., Ltd |
| Mifepristone | 84371-65-3 | Shanghai Canspec Scientific Instruments Co., Ltd |
| Glycyrrhizic acid 2NH4 | 80658-20-4 | Shanghai Canspec Scientific Instruments Co., Ltd |
| Lopinavir | 192725-17-0 | Shanghai Canspec Scientific Instruments Co., Ltd |
| Daidzein | 486-66-8 | Shanghai Canspec Scientific Instruments Co., Ltd |
| Clevidipine butyrate | 167221-71-8 | Shanghai Canspec Scientific Instruments Co., Ltd |
| Nevirapine | 129618-40-2 | Shanghai Canspec Scientific Instruments Co., Ltd |
| Lenalidomide | 191732-72-6 | Shanghai Canspec Scientific Instruments Co., Ltd |
| Dexamethasone | 50-02-2 | Shanghai Canspec Scientific Instruments Co., Ltd |
| Brexpiprazole | 913611-97-9 | Shanghai Canspec Scientific Instruments Co., Ltd |
| Meloxicam | 71125-38-7 | Shanghai Canspec Scientific Instruments Co., Ltd |
| Tapentadol Hydrochloride | 175591-09-0 | Shanghai Canspec Scientific Instruments Co., Ltd |
| Doxorubicin hydrochloride | 25316-40-9 | Beijing Sunflower and Technology Development Co., Ltd |
| Trandolapril | 87679-37-6 | Shanghai Canspec Scientific Instruments Co., Ltd |
| Dihydroartemisinin | 71939-50-9 | Shanghai Canspec Scientific Instruments Co., Ltd |
| Olmutinib | 1353550-13-6 | Shanghai Canspec Scientific Instruments Co., Ltd |
| Zopiclone | 43200-80-2 | Shanghai Canspec Scientific Instruments Co., Ltd |
| Flurbiprofen | 5104-49-4 | Shanghai Canspec Scientific Instruments Co., Ltd |
| Omeprazole | 73590-58-6 | Shanghai Canspec Scientific Instruments Co., Ltd |
| Losartan | 114798-26-4 | Shanghai Canspec Scientific Instruments Co., Ltd |
| Indometacin | 53-86-1 | Shanghai Canspec Scientific Instruments Co., Ltd |
| Enasidenib | 1446502-11-9 | Shanghai Canspec Scientific Instruments Co., Ltd |
| Valdecoxib | 181695-72-7 | Shanghai Canspec Scientific Instruments Co., Ltd |
| Axitinib | 319460-85-0 | Shanghai Canspec Scientific Instruments Co., Ltd |
| Voriconazole | 137234-62-9 | Beijing Sunflower and Technology Development Co., Ltd |
| PeraMpanel | 380917-97-5 | Shanghai Canspec Scientific Instruments Co., Ltd |
| AgoMelatine | 138112-76-2 | Beijing Sunflower and Technology Development Co., Ltd |
| Apatinib | 811803-05-1 | Shanghai Canspec Scientific Instruments Co., Ltd |
| Opemifene | 128607-22-7 | Shanghai Canspec Scientific Instruments Co., Ltd |
| Oxcarbazepine | 28721-07-5 | Shanghai Canspec Scientific Instruments Co., Ltd |
| Ornidazole | 16773-42-5 | Shanghai Canspec Scientific Instruments Co., Ltd |
| Tinidazole | 19387-91-8 | Shanghai Canspec Scientific Instruments Co., Ltd |
| Nimodipine | 66085-59-4 | Shanghai Canspec Scientific Instruments Co., Ltd |
| Amitriptyline hydrochloride | 549-18-8 | Beijing Sunflower and Technology Development Co., Ltd |
| Atorvastatin | 134523-00-5 | Shanghai Canspec Scientific Instruments Co., Ltd |
| Bupropion hydrochloride | 31677-93-7 | Beijing Sunflower and Technology Development Co., Ltd |
| Ketoconazole | 65277-42-1 | Shanghai Canspec Scientific Instruments Co., Ltd |
| Lumefantrine | 82186-77-4 | Shanghai Canspec Scientific Instruments Co., Ltd |
| Azithromycin | 83905-01-5 | Shanghai Canspec Scientific Instruments Co., Ltd |
| Venlafaxine hydrochloride | 99300-78-4 | Beijing Sunflower and Technology Development Co., Ltd |
| Aripiprazole | 129722-12-9 | Shanghai Canspec Scientific Instruments Co., Ltd |
| Ibrutinib | 936563-96-1 | Shanghai Canspec Scientific Instruments Co., Ltd |
| Cyclophosphamide | 50-18-0 | Shanghai Canspec Scientific Instruments Co., Ltd |
| Omarigliptin | 1226781-44-7 | Shanghai Canspec Scientific Instruments Co., Ltd |
| Brexpiprazole | 913611-97-9 | Shanghai Canspec Scientific Instruments Co., Ltd |
| Isavuconazole | 241479-67-4 | Shanghai Canspec Scientific Instruments Co., Ltd |
| Acyclovir | 59277-89-3 | Beijing Sunflower and Technology Development Co., Ltd |
| Tacrolimus | 104987-11-3 | Shanghai Canspec Scientific Instruments Co., Ltd |
| Saxagliptin hydrochloride | 709031-78-7 | Beijing Sunflower and Technology Development Co., Ltd |
| Lidocaine | 137-58-6 | Shanghai Canspec Scientific Instruments Co., Ltd |
| Ropivacaine hydrochloride | 132112-35-7 | Shanghai Canspec Scientific Instruments Co., Ltd |
| Bupivacaine hydrochloride | 14252-80-3 | Shanghai Canspec Scientific Instruments Co., Ltd |
| Carbamazepine | 298-46-4 | Shanghai Canspec Scientific Instruments Co., Ltd |
| Terfenadine | 50679-08-8 | Shanghai Canspec Scientific Instruments Co., Ltd |
| Mestranol | 72-33-3 | Shanghai Canspec Scientific Instruments Co., Ltd |
| Diphenhydramine Hydrochloride | 147-24-0 | Shanghai Canspec Scientific Instruments Co., Ltd |
| Captopril | 62571-86-2 | Shanghai Canspec Scientific Instruments Co., Ltd |
| Erythromycin | 114-07-8 | Shanghai Canspec Scientific Instruments Co., Ltd |
| Valsartan | 137862-53-4 | Shanghai Canspec Scientific Instruments Co., Ltd |
| Gefitinib | 184475-35-2 | Shanghai Canspec Scientific Instruments Co., Ltd |
| Sunitinib | 557795-19-4 | Shanghai Canspec Scientific Instruments Co., Ltd |
| Bosentan | 147536-97-8 | Shanghai Canspec Scientific Instruments Co., Ltd |
| Darusentan | 171714-84-4 | Shanghai Canspec Scientific Instruments Co., Ltd |
| Pentobarbital sodium | 57-33-0 | Shanghai Canspec Scientific Instruments Co., Ltd |
| Famciclovir | 104227-87-4 | Shanghai Canspec Scientific Instruments Co., Ltd |
| Ondansetron | 99614-02-5 | Shanghai Canspec Scientific Instruments Co., Ltd |
| Ritonavir | 155213-67-5 | Shanghai Canspec Scientific Instruments Co., Ltd |
| Clarithromycin | 81103-11-9 | Shanghai Canspec Scientific Instruments Co., Ltd |
| Ivabradine | 155974-00-8 | Shanghai Canspec Scientific Instruments Co., Ltd |
| Cetirizine hydrochloride | 83881-52-1 | Shanghai Canspec Scientific Instruments Co., Ltd |
| 5-Fluorouracil | 51-21-8 | Shanghai Canspec Scientific Instruments Co., Ltd |
| ciprofloxacin hydrochloride | 93107-08-5 | Beijing Sunflower and Technology Development Co., Ltd |
| Acetaminophen | 103-90-2 | Shanghai Canspec Scientific Instruments Co., Ltd |
| Granisetron hydrochloride | 107007-99-8 | Shanghai Canspec Scientific Instruments Co., Ltd |
| Entrectinib | 1108743-60-7 | Shanghai Canspec Scientific Instruments Co., Ltd |
| Fluoxetine hydrochloride | 56296-78-7 | Beijing Sunflower and Technology Development Co., Ltd |
| Furazolidone | 67-45-8 | Beijing Sunflower and Technology Development Co., Ltd |
| Fluconazole | 86386-73-4 | Shanghai Canspec Scientific Instruments Co., Ltd |
| Glipizide | 29094-61-9 | Beijing Sunflower and Technology Development Co., Ltd |
| Posaconazole | 171228-49-2 | Shanghai Canspec Scientific Instruments Co., Ltd |
| Dapoxetine hydrochloride | 129938-20-1 | Beijing Sunflower and Technology Development Co., Ltd |
| Metformin hydrochloride | 15537-72-1 | Shanghai Canspec Scientific Instruments Co., Ltd |
| Glibenclamide | 10238-21-8 | Shanghai Canspec Scientific Instruments Co., Ltd |
| Glimepiride | 93479-97-1 | Shanghai Canspec Scientific Instruments Co., Ltd |
| Flutrimazole | 119006-77-8 | Shanghai Canspec Scientific Instruments Co., Ltd |
| Isopropiram Fumarate | 84873-04-1 | Shanghai Canspec Scientific Instruments Co., Ltd |
| Procainamide hydrochloride | 614-39-1 | Shanghai Canspec Scientific Instruments Co., Ltd |
| Loratadine | 79794-75-5 | Tianjin xiensi Biochemical Technology Co., Ltd |
| Ixazomib | 1072833-77-2 | Shanghai Canspec Scientific Instruments Co., Ltd |
| Meropenem | 96036-03-2 | Shanghai Canspec Scientific Instruments Co., Ltd |
| Ribavirin | 36791-04-5 | Tianjin xiensi Biochemical Technology Co., Ltd |
| Ramelteon | 196597-26-9 | Shanghai Canspec Scientific Instruments Co., Ltd |
| Lansoprazole | 103577-45-3 | Shanghai Canspec Scientific Instruments Co., Ltd |
| Clonidine hydrochloride | 4205-91-8 | Shanghai Canspec Scientific Instruments Co., Ltd |
| Curcumin | 458-37-7 | Shanghai Canspec Scientific Instruments Co., Ltd |
| Rivaroxaban | 366789-02-8 | Shanghai Canspec Scientific Instruments Co., Ltd |
| Metyrapone | 54-36-4 | Shanghai Canspec Scientific Instruments Co., Ltd |
| Chlorpropamide | 94-20-2 | Shanghai Canspec Scientific Instruments Co., Ltd |
| Candesartan | 139481-59-7 | Shanghai Canspec Scientific Instruments Co., Ltd |
| Clonazepam | 1622-61-3 | Shanghai Canspec Scientific Instruments Co., Ltd |
